# Development of 11-Plex MOL-PCR Assay for the Rapid Screening of Samples for Shiga Toxin-Producing *Escherichia coli*

**DOI:** 10.3389/fcimb.2016.00092

**Published:** 2016-08-31

**Authors:** Travis A. Woods, Heather M. Mendez, Sandy Ortega, Xiaorong Shi, David Marx, Jianfa Bai, Rodney A. Moxley, T. G. Nagaraja, Steven W. Graves, Alina Deshpande

**Affiliations:** ^1^Department of Chemical and Biological Engineering, University of New MexicoAlbuquerque, NM, USA; ^2^The New Mexico ConsortiumLos Alamos, NM, USA; ^3^Translational Biomedical Sciences, University of RochesterRochester, NY, USA; ^4^Department of Diagnostic Medicine/Pathobiology, College of Veterinary Medicine, Kansas State UniversityManhattan, KS, USA; ^5^Department of Statistics, University of Nebraska-LincolnLincoln, NE, USA; ^6^School of Veterinary Medicine and Biomedical Sciences, University of Nebraska-LincolnLincoln, NE, USA; ^7^Los Alamos National Laboratory, Analytics, Intelligence and Technology DivisionLos Alamos, NM, USA

**Keywords:** STEC, MOL-PCR, multiplex PCR, Shiga toxin, EHEC

## Abstract

Strains of Shiga toxin-producing *Escherichia coli* (STEC) are a serious threat to the health, with approximately half of the STEC related food-borne illnesses attributable to contaminated beef. We developed an assay that was able to screen samples for several important STEC associated serogroups (O26, O45, O103, O104, O111, O121, O145, O157) and three major virulence factors (*eae, stx*_1_*, stx*_2_) in a rapid and multiplexed format using the Multiplex oligonucleotide ligation-PCR (MOL-PCR) assay chemistry. This assay detected unique STEC DNA signatures and is meant to be used on samples from various sources related to beef production, providing a multiplex and high-throughput complement to the multiplex PCR assays currently in use. Multiplex oligonucleotide ligation-PCR (MOL-PCR) is a nucleic acid-based assay chemistry that relies on flow cytometry/image cytometry and multiplex microsphere arrays for the detection of nucleic acid-based signatures present in target agents. The STEC MOL-PCR assay provided greater than 90% analytical specificity across all sequence markers designed when tested against panels of DNA samples that represent different STEC serogroups and toxin gene profiles. This paper describes the development of the 11-plex assay and the results of its validation. This highly multiplexed, but more importantly dynamic and adaptable screening assay allows inclusion of additional signatures as they are identified in relation to public health. As the impact of STEC associated illness on public health is explored additional information on classification will be needed on single samples; thus, this assay can serve as the backbone for a complex screening system.

## Introduction

Shiga toxin-producing *E. coli* (STEC) is an important foodborne pathogen that caused an estimated 2.8 million illnesses, 3890 cases of acute kidney failure (hemolytic uremic syndrome) (HUS), and 230 deaths worldwide in 2012 (Majowicz et al., 2014). In the United States, STEC O157:H7 is responsible for the majority of these cases, and caused an estimated 4928 human infections per year from 2003 to 2012 (Heiman et al., 2015). In addition to more severe manifestations of infection like HUS, human infections can also be as mild as watery diarrhea progressing to hemorrhagic colitis (HC), manifested clinically as bloody diarrhea. Ingestion of food contaminated with STEC is one of the pathways to human STEC infection. The two most common food sources of STEC infection are beef products and leafy vegetables (1144 and 922 reported illnesses, respectively, from 2003 to 2012) (Heiman et al., 2015). STEC O157:H7, shed in cattle feces, is a major source of environmental contamination, including that of waterways near feedlots. Vegetable cultivation and processing operations utilizing these fecal contaminated waterways lead to points of STEC contamination for leafy greens and other produce (Cooley et al., 2014; Heiman et al., 2015). In addition to STEC O157:H7, infections with non-O157:H7, and in particular serogroups O26, O45, O103, O111, O121, O145 increased in the U.S. during 2000–2010 (Gould et al., 2013). Further, between May-July 2011, outbreaks of HC and HUS occurred in Europe due to a newly emergent STEC serotype identified as O104:H4 (European Food Safety Authority, 2011; Grad et al., 2012). While STEC serotype O104:H4 has not been found in cattle in the U.S. (Shridhar et al., 2016) and its reservoir has not been determined, it still presents a threat to human health. Thus, within a short span of time, a significant number of serogroups of concern have been identified.

Shiga toxin (Stx), also known as Vero cytotoxin and formerly as Shiga-like toxin, is produced by STEC as one of two antigenically distinct types, Stx1 or Stx2, encoded, respectively, by *stx*_1_ and *stx*_2_ (Scheutz et al., 2012; Melton-Celsa, 2014). Within Stx1 and Stx2, subtypes representing amino acid sequence variants with varying types or levels of toxicity, biological activity, and immunological distinction are recognized (Scheutz et al., 2012; Melton-Celsa, 2014). Stx inhibits protein synthesis in susceptible host cells and species, e.g., endothelial cells in humans (Ferens and Hovde, 2011). The subtypes of Stx1 include Stx1a, Stx1c, Stx1d; those of Stx2 include Stx2a, Stx2b, Stx2c, Stx2d, Stx2e, Stx2f, and Stx2g. Using this nomenclature, the toxins originally named Stx1 and Stx2 are Stx1a and Stx2a, encoded by *stx*_1a_ and *stx*_2a_, respectively.

STEC strains, by definition, produce Stx or at least carry *stx*, but those causing disease in human patients have additional virulence factors required for pathogenesis. A subset of STEC known as enterohemorrhagic *E. coli* (EHEC) are positive for both *stx* and *eae*, or are *stx*-positive but *eae*-negative and yet have been isolated from patients with hemorrhagic colitis and/or HUS (Croxen et al., 2013). The *eae* gene encodes for intimin, the protein responsible for intimate attachment to the host cell membrane through its binding to the translocated intimin receptor (Croxen et al., 2013). Those EHEC strains lacking *eae* colonize the human intestine via adhesins other than intimin (Croxen et al., 2013). STEC classification into serogroups is provided by O (lipopolysaccharide) and H (flagellar) antigens. EHEC strains of the seven major serogroups that cause disease in the U.S. (i.e., O26, O45, O103, O111, O121, O145, and O157) have been classified as adulterants in raw, non-intact beef by the U.S. Department of Agriculture, Food Safety and Inspection Service (FSIS) (Stromberg et al., 2016). In the present study, we will refer to EHEC of these seven serogroups together with O104:H4 as STEC-8.

The current state of the art in molecular diagnostics for STEC-8 detection is multiplex PCR (real time or end point). This assay chemistry identifies STEC serogroups by detecting unique DNA sequences for genes expressed in the O-antigen gene clusters of the genomic DNA (Perelle et al., 2004; DebRoy et al., 2011; Bai et al., 2012). Multiplex real-time PCR (Noll et al., 2015) detection assays are able to detect a variety of genes that determine serogroup and virulence in a single sample (Wang et al., 2002), but are limited to a single reaction multiplex level of 3–4 unique sequences. In contrast, higher level end point multiplex-PCR single reaction nucleic acid detection assays have sample throughput limitations due to constraints of analysis technology (pulse field gel electrophoresis, capillary electrophoresis) (Bosilevac and Koohmaraie, 2011; Paddock et al., 2013). Multiplexed oligonucleotide PCR (MOL-PCR), a nucleic acid-based assay chemistry capable of the simultaneous detection of several specific DNA signatures in a high-throughput single reaction using a multiplex ligation technique, addresses limitation of both plex level and throughput. MOL-PCR: (1) does not require multiple rounds of analysis; (2) has minimal setup and operational costs; (3) is capable of both pathogen identification and provides additional characterization; and (4) is easily scalable in high-throughput situations (Deshpande et al., 2010). The major objective of this study was to develop an assay utilizing high-throughput MOL-PCR to identify all of the STEC-8 serogroups while also detecting *stx*_1_*, stx*_2_, and *eae* in a single sample and in a single reaction. We describe herein, the development and validation of this assay using blinded and known panels of DNA samples that represented the STEC-8 serogroups and various toxin gene profiles. This STEC-8 MOL-PCR assay was highly multiplexed, but more importantly the underlying chemistry also makes it dynamic and adaptable to allow inclusion of additional signatures (serogroups, toxins, etc.) as they are identified as being of interest to public health. As the impact of STEC associated illness on public health is explored additional information on classification will be needed on single samples; this assay can serve as the backbone for a complex screening system.

## Materials and methods

### MOL-PCR signature selection

The STEC-8 MOL-PCR assay was designed from probes developed for multiplex PCR to detect O26, O45, O103, O111, O121, O145, and O157:H7 serogroups as well as STEC and EHEC virulence genes (*stx*_1,_*stx*_2_, and *eae)* (Bai et al., 2012; Paddock et al., 2012). In that work, searches were conducted using BLASTN to generate forward and reverse primers unique to each virulence marker: *stx*_1_, *stx*_2_, and *eae* (Bai et al., 2012). The primers for *stx*_1_ were designed to detect a sequence common to *stx*_1a_, *stx*_1c_, and *stx*_1d_, and validated to detect all three subtypes (Bai et al., 2012). The primers for *stx*_2_ were designed and *in-silico* validated to detect a sequence common to all *stx*_2_ subtypes but *stx*_2f_, and experimentally validated to detect *stx*_2a_, *stx*_2b_, *stx*_2c_, and *stx*_2d_, which were the only subtypes available in the collection (Bai et al., 2012).

In our work, multiplex PCR primer sequences for all serogroups as well as virulence genes were searched against all available genomes in GenBank using BLASTN (blast.ncbi.nlm.nih.gov). This search was done as a first step to verify that this section of sequence was truly unique and able to confer serogroup specificity. Both forward and reverse sets of primers for each sequence did not return homology outside of their expected serogroup specific sequences. In most cases, the forward primer sequence for each marker from the previous multiplex PCR (Bai et al., 2012; Paddock et al., 2012) work was used as the basis for probe (MOLigo pair) design for the MOL-PCR assay.

#### MOLigo pair design

MOLigos were designed using MOLigoDesigner developed at Los Alamos National Laboratory (LANL) (Gans and Wolinsky, 2008). The tool generates the sequence design for two single stranded MOLigos (MOLigo-1 and MOLigo-2) for each unique sequence being targeted. The MOLigo detection event requires the annealing of two MOLigos adjacent to each other, on the target DNA. MOLigo-2 included a universal reverse primer sequence (5′-ACTCGTAGG GAATAAACCGT-3′), followed by a microsphere specific anti-TAG 24-mer sequence and the site-specific STEC target DNA sequence on the 3′ end with a total nucleotide length varying between 61 and 74 bases. MOLigo-1 was synthesized with a phosphorylation tag at its 5′ end followed by the site-specific STEC DNA sequence and a universal forward primer sequence (5′-TCTCACTTC TTACTACCGCG-3′) with a varying total nucleotide length between 36 and 55 bases (Table 1). The total amplicon (fully ligated MOLigo-1 to MOLigo-2) length per target varied between 101 and 127 bases with the site-specific region being between 37 and 63 bases on the target sequence. MOLigos were designed to be complementary to the desired conserved sequences in the O-antigen gene cluster unique to each serogroup of the STEC-8. Each MOLigo pair was designed as the reverse complement of a unique contiguous sequence previously identified as serogroup-specific for each of the STEC-8. The assay was also designed to detect conserved sequences for *stx*_1_, *stx*_2_, and *eae* at the same time as the STEC-8 serogroups.

**Table 1:** **Sequences for MOLigo pairs with all portions defined**.

**Serogroup/gene^a^**	**xTAG^b^**	**ID^c^**	**Sequence^d^**
O26		rNO26Fwzx958A(+)M1	Phos-ACCCACCCCCCCTAAACT**TCTCACTTCTTACTACCGCG**
	A034	rNO26Fwzx958A(+)M2	*ACTCGTAGGGAATAAACCGT*tgatatagtagtgaagaaataagtGATACTTTGAACCTTATATCCCAATATAGT
O45		NO45Fwzx377G(+)M1	Phos-TGGACAGCCCACTTGCAG**TCTCACTTCTTACTACCGCG**
	A065	NO45Fwzx377G(+)M2	*ACTCGTAGGGAATAAACCGT*tgagtaagtttgtatgtttaagtaGCCAAACCAACTATGAACTGTC
O103		NO103Fwzx303G(+)M1	Phos-CCCGTACTTATAATAAAACAACAGGC**TCTCACTTCTTACTACCGCG**
	A038	NO103Fwzx303G(+)M2	*ACTCGTAGGGAATAAACCGT*agtaagtgttagatagtattgaatTCTGATATTTTACTGGAAAAAAGCACC
O104		M1O104-b62-wzx821G	Phos-AATAAAACCTGCGATATCTGCT**TCTCACTTCTTACTACCGCG**
	A062	M2O104-b62-wzx821G	*ACTCGTAGGGAATAAACCGT*tgaaatgtgtatttgtatgtttagGTTGAAATTCTTTGCGCGAC
O111		JBO111Fwzx496C(+)M1	Phos-CACTCTTGTAATTACTTCAAAAAAACATGAT**TCTCACTTCTTACTACCGCG**
	A046	JBO111Fwzx496C(+)M2	*ACTCGTAGGGAATAAACCGT*gtgattgaatagtagattgtttaaGCCATATATTACTATAGAAGCCCAGAG
O121		NO121Fwzx420T(+)M1	Phos-AATATAATGATGAATCTAAGCGTTGTTATAAAAAT**TCTCACTTCTTACTACCGCG**
	A027	NO121Fwzx420T(+)M2	*ACTCGTAGGGAATAAACCGT*aagatgatagttaagtgtaagttaAGTATAACCTTTTACTTTCATGACAGGA
O145		M1O145b35Fwzx98T	Phos-AAAGTCGAGCAAGCAAAACA**TCTCACTTCTTACTACCGCG**
	A035	M2O145b35Fwzx98T	*ACTCGTAGGGAATAAACCGT*aataagagaattgatatgaagatgCACTCCTAAATCTGTTGATGGTA
O157:H7		NO157FECs2841-578G(+)M1	Phos-CACCTTCACCTGTAGTAATAGTTTTATTT**TCTCACTTCTTACTACCGCG**
	A028	NO157FECs2841-578G(+)M2	*ACTCGTAGGGAATAAACCGT*gatagatttagaatgaattaagtgTGTCATTCGTGACAACCATTC
*stx*_1_		M1stx1-b45-626A	Phos-CATCCAGTGTTGTACGAAATCC**TCTCACTTCTTACTACCGCG**
	A045	M2stx1-b45-626A	*ACTCGTAGGGAATAAACCGT*gttagttatgatgaatattgtgtaATAAGAACGCCCACTGAGAT
*stx*_2_		M1stx2-b19-565C	Phos-GACAGCAGTTATACCACTCTG**TCTCACTTCTTACTACCGCG**
	A019	M2stx2-b19-565C	*ACTCGTAGGGAATAAACCGT*gtgtgttatttgtttgtaaagtatCGGTTTCCATGACAACG
*eae*		eae2120A(+)M1	Phos-TGGTCAGGTCGGGGCG**TCTCACTTCTTACTACCGCG**
	A056	eae2120A(+)M2	*ACTCGTAGGGAATAAACCGT*aattagaagtaagtagagtttaagTTCCGAAAACATGCTGGCATT
Universal Forward		DualBiotin Univ Forwd Primer	Dual-Biotin-**CGCGGTAGTAAGAAGTGAGA**
Universal Reverse		Universal Reverse Primer	ACTCGTAGGGAATAAACCGT

a Serogroup and virulence marker of MOLigo pair.

b MagPlex-TAG microsphere with anti-TAG sequence.

c MOLigo probe identification name.

d Target hybridizing sequence (underlined and uppercase), universal forward primer sequence (bold and uppercase), universal reverse primer sequence (italic and uppercase), TAG sequence (lowercase). Phos is 5′ phosphorylation. Dual-Biotin is a 5′ Dual Biotin label.

MOLigoDesigner uses the nearest-neighbor model for calculating the melting temperature (T_m_) of hybridized oligonucleotides. The following inputs were used during MOLigo pair design; ligation T_m_ = 55°C, STEC DNA specific sequence = 12–45 base pairs, with a maximum Delta T_m_ = 5°C, oligonucleotide concentration = 5 nM, monovalent salt concentration = 60 mM, divalent salt concentration = 2 mM, and nucleotide triphosphates concentration = 2 μM. During the design process the various tag sequences and primer sequences were added to either appropriate MOLigo partner as described previously.

Once designed, MOLigo pairs were *in-silico* tested against all-available genomes in NCBI's BLAST program to look for possible interactions of MOLigos with non-target genome sequences. All MOLigo pairs that returned low non-target interactions (*E* > 0.002) were then considered to be unique probes for STEC-8 associated DNA sequences. These MOLigo pair sequences were then tested using NUPACK (NUPACK.org) (Zadeh et al., 2011) to evaluate interactions between various MOLigo-1/MOLigo-2 pairings, primer-dimers reactions. No interactions were found with a free energy lower than -2.5 kcal/mol at 50°C and were therefore considered to be non-interacting. This check ensured that the MOLigos could be used in a multiplex reaction and were compatible.

Integrated DNA Technologies (IDT DNA) (Coralville, IA) synthesized all finalized MOLigo pairs on a 25–100 nmole scale, depending on oligonucleotide length, with standard desalting as the purification method. IDT DNA also synthesized the universal forward and reverse primers as well as the full-length control consisting of the full length STEC serogroup O26 MOLigo-1-MOLigo-2 pair synthesized as one unit with a unique TAG sequence, which was used as a positive control for PCR amplification. All custom oligonucleotides were reconstituted in TE buffer (10 mM Tris, pH 7.5, and 1 mM EDTA, Affymetrix; Santa Clara, CA) at concentrations of 20 μM–200 μM. MOLigos and primers were stored at −20°C for long-term stability.

### MOLigo ligation to template DNA

Discrimination of the STEC-8 relevant DNA sequences was accomplished during the first step, ligation. The ligation reaction was carried out with either PFU DNA (Agilent Technologies; Santa Clara, CA) ligase, or Ampligase DNA ligase (Epicentre; Madison, USA) as they are both thermostable ligases. The ligation reaction volume was 10 μL for capped tube reactions and 20 μL for PCR plate (USA Scientific; Orlanodo, FL) reactions sealed with TempPlate sealing film (USA Scientific). Regardless of volume, the final reaction contained 1X Ligase buffer (Epicentre or Agilent Technologies), individual MOLigos at 4 nM, sheared UltraPure™ Salmon Sperm DNA (ThermoFisher, Grand Island, NY) at 0.15 mg/mL, and DNA Ligase at 1.25 units (U) per reaction. Uracil-DNA Glycosylase (ThermoFisher) (UDG) (5 U/μl) was added at 2.5 U per reaction to control for amplicon carry-over from previous experiments. (Longo et al., 1990) The sealed tubes or plates were run on a thermal cycler (Applied Biosystems, Foster City, CA) with the following conditions: 37°C for 30 min followed followed by 3 min at 95°C and then 30 cycles of 25 s (s) at 95°C and 50°C for 2 min. The reaction was held at 95°C for 30 min at the end to inactivate the UDG before holding at 4°C.

The ligation step, including addition of all non-target DNA, buffers, and enzymes, was performed inside a PCR hood or dead air box to prevent any amplicon or target STEC DNA from contaminating reagent stocks.

### Amplification of detection event

PCR amplification of the ligation product was performed using a standard PCR amplification protocol. Briefly, 2 μL of the ligation product was transferred to tubes or a PCR plate containing 8 μL or 18 μL of PCR master mix. The PCR final reaction contained of 1X Amplitaq Gold buffer (Applied Biosystems; Foster City, CA), 4 mM MgCl_2_, 0.5 μM universal forward primer, 0.1 μM universal reverse primer, dUTP mix (ThermoFisher, Grand Island, NY) (200 μM A, C, G and 400 μM U) to work in conjunction with the UDG in the previous step, and Amplitaq Gold DNA polymerase (Applied Biosystems; Foster City, CA) at 0.5 U/reaction. The PCR amplification was performed in a thermocycler with the following conditions: 95°C for 10 min followed by 45 cycles: 95°C for 15 s, 58°C for 20 s, and 72°C for 20 s. A 7-min extension at 72°C was added after the cycles with a final hold at 4°C until further processing.

The addition of the ligation product and any control DNA (purified or synthetic) to the PCR plate was performed in a separate hood from the hood where the master mix was dispensed.

As an additional control for amplicon contamination, the pre-PCR/ligation laboratory was not re-entered until the next day once the amplification thermocycling was started in the post-PCR laboratory. During the post-processing of the amplified samples there is a high probability of amplicon aerosol generation, which is easily carried over between laboratories. In implementing this one-way workflow, contamination from the high quantity of amplified MOLigo amplicons during PCR was greatly reduced. One of the goals of this research was to not only develop an assay for STEC detection, but also to develop a standardized protocol for implementing MOL-PCR based detection of STEC for use in public health/testing laboratories.

### Capture of amplified product to microsphere array

Hybridization of the amplified product to the anti-tag bearing Luminex MagPlex-TAG™ (Luminex Corp.; Austin, TX) microspheres was done using a mix of 13 different microspheres; one for each MOLigo product (STEC-8 along with *stx*_1_, *stx*_2_, and *eae*) as well as an assay positive control and an additional microsphere that served as either a blank or PCR positive control. The microsphere mix was made by combining 36 μL of each MagPlex-TAG™ microsphere stock in a microcentrifuge tube, pelleting the microspheres at 7500 × G for 5 min followed by suspending the pellet in 900 μL of 800 mM NaCl/50 mM MES buffer (Fisher Bioreagents). Ten microliters of this solution was added to each well of the PCR product plate giving 1000 microspheres for each region (13,000 microspheres total) per reaction well on the plate. The total volume for the hybridization reaction was 30 μL; 20 μL PCR product and 10 μL microsphere mix. Hybridization was carried out on a thermal cycler block with a slow ramp down from 95°C (95°C for 3 min (min), 85°C for 1 min, 75°C for 1 min, 65°C for 1 min, 55°C for 1 min, 50°C for 30 min, 45°C for 1 min, 40°C for 1 min, 35°C for 1 min, 30°C for 1 min and hold at 25°C).

### Fluorescent reporter labeling of complex and analysis

Before analysis on the Luminex® 100™/MAGPIX®, the samples were placed on a magnetic plate (EMD Millipore; Billerica, MA) for 5 min before being inverted sharply to remove supernatant. The microsphere pellets in each well were then suspended in 25 μL of 10 μg/mL Streptavidin Phycoerythrin (SAPE) (BD Biosciences; San Jose, CA) in TE buffer and incubated at room temperature for 30 min, protected from light. Following incubation with the SAPE, the plate was pelleted again on the magnetic plate as stated above and the pellet was suspended in 100 μL of TE buffer and transferred to a standard 96-well plate for analysis.

The 96-well plate was analyzed on a Luminex® 100™ or MAGPIX®, recording the median fluorescence intensity (MFI) values for each region with a lower limit of 100 events per region. Included on the plate was a column of eight no-template controls to account for any cross reactivity of the MOLigo pairs in the absence of target DNA. Also included on the plate was a set of 8 process controls consisting of DNA isolates from each STEC-8 serogroup. These MFI values for the no-template controls and samples were used as the basis to make a call for “positive” or “negative” for each target DNA sequence. An exact Wilcoxon rank sum analysis was done between the eight independent no-template controls and the three replicates of each sample being tested.

### Statistical analysis

The small sample sizes used in the assay per test (8 no-template controls and 3 replicates) were appropriate for applying an exact Wilcoxon rank-sum statistical test for making “positive” or “negative” determinations. Non-parametric tests, such as these are of value in cases where data sets are small, there are non-matched pairs, and little is known of the distribution of the data (Whitley and Ball, 2002; Walker and Institute, 2010). Wilcoxon rank sum was performed by 3 steps, which were automated in this assay in an Excel macro.

### STEC-8 MOL-PCR assay optimization

A significant amount of care was used to design the probes for detecting STEC-8, which relied initially on work done by Bai et al. (2012) in their multiplex PCR assay development. In most of that work the *wzx* (O-antigen-flippase) genes in the O-antigen (lipopolysaccharide) gene cluster were targeted for O26, O45, O103, O104, O111, O145, and *wbqE* (encodes for putative glycosyl transferase) and *wbqF* (encodes for putative acetyl transferase) for O121. STEC O157 was detected using the *rfbE* gene, as it identifies the O157 serogroup. Initially each MOLigo pair was tested individually on its respective serogroup reactive strain to develop the appropriate ligation and amplification protocol. Each of these strains also has an associated virulence profile relative to the specific toxin gene sequences tested in this assay; *stx*_1_, *stx*_2_, and *eae*.

During early testing and validation, each assay product was divided such that half of the product was visualized on an agarose gel stained with ethidium bromide and the second half was hybridized to Luminex microspheres. MOLigoDesigner produced a MOLigo pair that consisted of a positive (+) strand MOLigo pair as well as a negative (−) strand (reverse complement) MOLigo pair. Computational analysis of predicted MOLigo strands indicated the absence of interactions between strands, therefore during the protocol optimization step each set (positive or negative) was evaluated individually against their complement target DNA from ATCC. STEC serogroup samples from ATCC were used at 5 × 10^6^ copies of DNA per reaction. MOLigos were successfully evaluated against their respective ATCC serogroup by titrating the respective pairs of MOLigos from 1 × 10^7^ copies down to 1 × 10^4^ genomic copies of template DNA.

**Figure 1:**
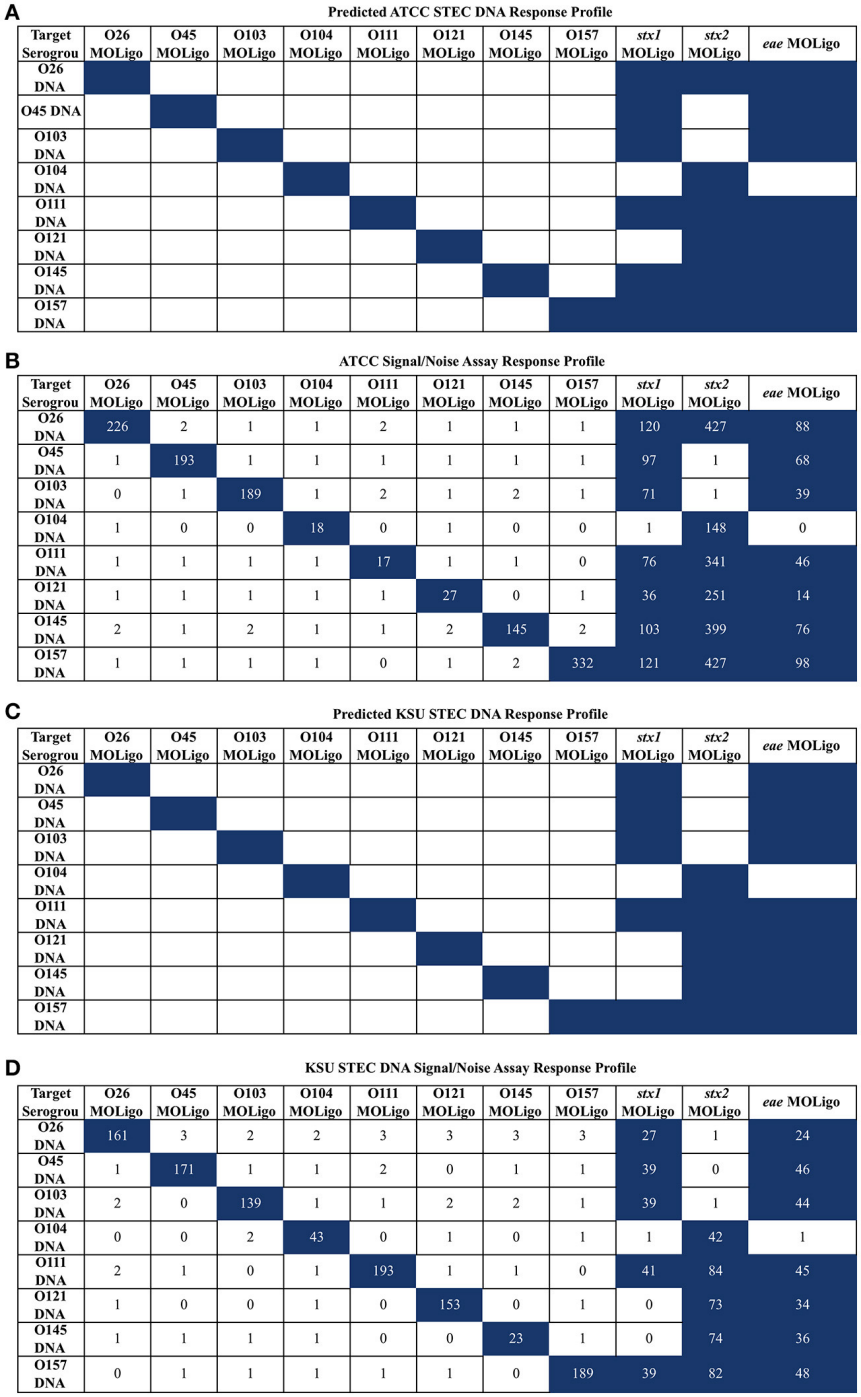
**STEC-8 MOL-PCR assay evaluated against commercial STEC associated DNA (ATCC) and STEC associated DNA from laboratory cultures (KSU), reported as signal to noise ratio of median fluorescence intensity**. Values were rounded to the nearest integer where zero values were from reported zero median fluorescence intensity; predicted highest and assay highest values highlighted in blue. **(A)** ATCC STEC DNA predicted interactions agreed with the **(B)** corresponding assay results, with the exception of O121 (BAA-2219D) *stx*_1_ reporting as present when known not to be. **(C)** KSU STEC DNA predicted interactions were in complete agreement with the assay results **(D)** for the KSU samples.

To confirm the specificity of the STEC-8 MOL-PCR assay, it was tested against two sets of STEC-8 samples, ATCC and KSU. Both sets contained all STEC-8 serogroups, and had slightly different virulence gene profiles. In these tests, the full set of all 11 MOLigo pairs were combined to perform the fully multiplexed assay. Figure 1 displays the integer signal-to-noise ratio of median fluorescence intensity reported for each STEC DNA sample for each MOL-PCR probe compared to the known profile. There was agreement between the known and MOL-PCR reported profiles for both sets of samples tested, except ATCC STEC O121 (BAA-2219D) reported as being positive for *stx*_1_ by MOL-PCR when it was known to be negative for that virulence gene.

### *E. coli* and non-*E. coli* DNA used for assay validation

The MOL-PCR STEC-8 assay was evaluated using multiple panels of DNA samples. Initial development was done on two independent sets of DNA: samples obtained from the American Type Culture Collection (ATCC) and samples obtained from Kansas State University (KSU). Template DNA obtained from ATCC (Manassas, VA) included the following: BAA-460D (O157:H7), BAA-2192D (O145:NM), BAA-2193D (O45:H2), BAA-2196D (O26:H11), BAA-2215D (O103:H11), BAA-2217D (O146 mislabeled as O111 by ATCC), BAA-2219D (O121:H19), BAA-2326D (O104:H4), BAA-2440D (O111). This DNA was well characterized, purified, and lyophilized. KSU sample strains were H30 (O26), CDC 96-3285 (O45), CDC 90-3128 (O103), ATCC BAA-2326 (O104), JB1-95 (O111), CDC 97-3068 (O121), 83-75 (O145), and 380-94 (O157).

The first phase of testing was conducted on a set of 99 reference purified DNA (purified DNA) samples prepared in the laboratory of R. Moxley, which contained O26 (10), O45 (10), O103 (10), O104 (9), O111 (10), O121 (10), O145 (10), O157 (10) across a range of H (flagellar) antigens expressing mixed combinations of virulence genes. This set of DNA represents a mix of isolates from both human and bovine fecal samples. In addition to samples that were STEC-8, there were 6 samples that were O antigen classified as O101, O8, O142, O150, O104 (non-STEC), and O124. Also included in this set were 14 samples that were STEC unrelated, used here as an exclusivity panel: *Aeromonas hydrophila, Enterobacter cloacae, Enterococcus faecalis, Klebsiella pneumoniae, Morganella morganii, Proteus mirabilis, Proteus vulgaris, Providencia rettgeri, Pseudomonas aeruginosa, Serratia marcescens, Staphylococcus aureus, Yersinia enterocolitica, Streptococcus gallolyticus*, and *Salmonella* Typhimurium. This exclusivity panel is a representative collection of related organisms that are commonly found in fecal samples and serve as an appropriate analog for interactions of the STEC-8 MOL-PCR with non-target genomic DNA (non-reactive). The second phase of testing was conducted on a panel of 144 DNA samples that were blinded and provided by the T. Nagaraja laboratory. These represented isolates from a diverse set of samples obtained from human infants, male and female adult humans, rabbit, bovine (cow and calf), and cattle feed. For all tests the complete STEC-8 MOL-PCR 11-plex was used.

## Results

### Evaluation of reference panel

The reference samples were analyzed with the STEC-8 MOL-PCR assay and used to train the exact Wilcoxon algorithm for making semi-automated calls for the serogroup and virulence gene profile. The exclusivity panel was used to develop the thresholds for determining the positivity of a test sample. The mean signal-to-noise ratio for each sample, across all markers, was calculated and used as a basis to adapt the exact Wilcoxon rank sum test.

#### Evaluation of exclusivity panel

**Figure 2:**
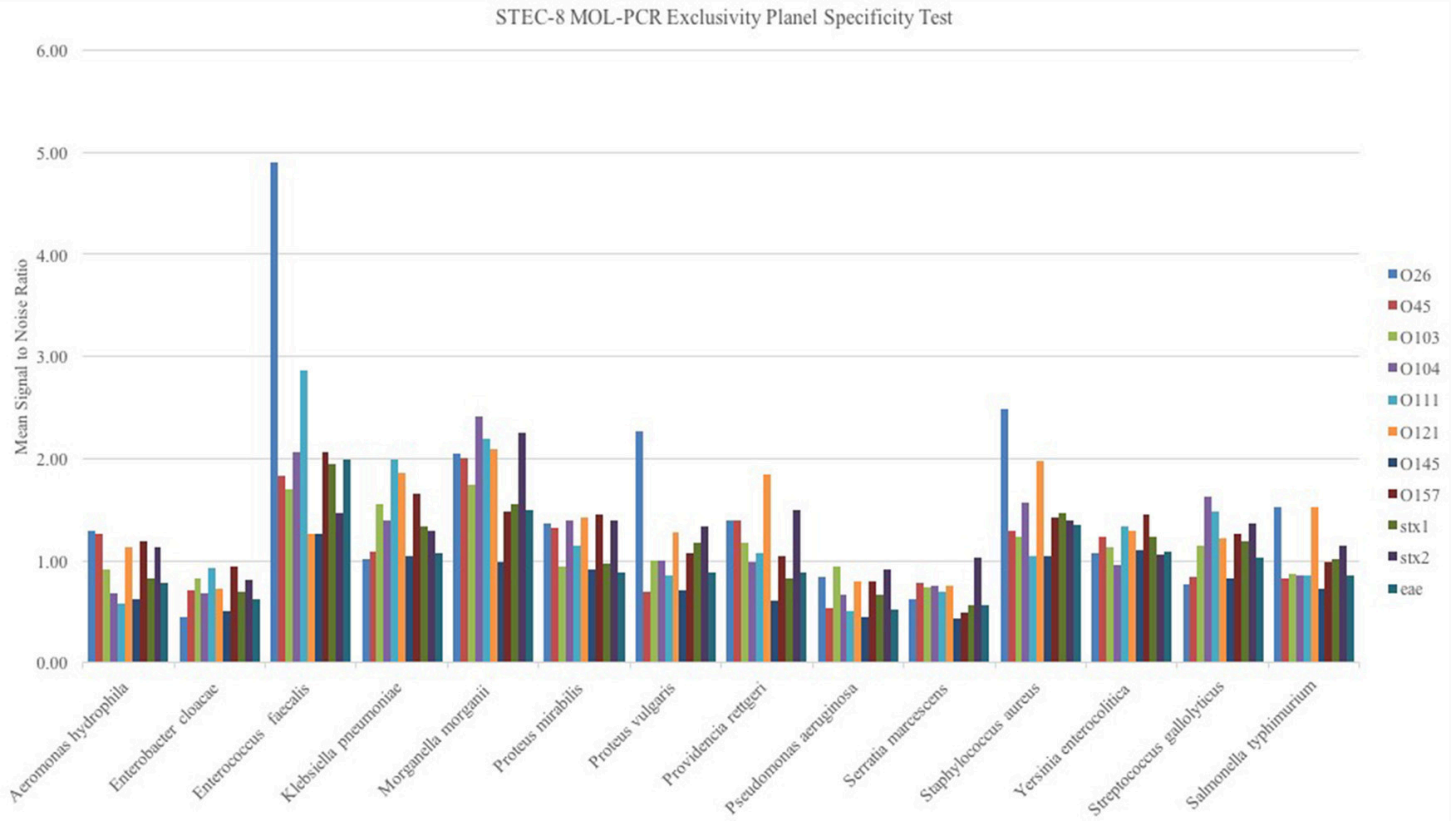
**Exclusivity panel of STEC unrelated DNA samples from bacterium as noted in figure evaluated with the STEC-8 MOL-PCR assay**. The evaluation 11-plex is color coded on the figure with corresponding signal-to-noise ratio being shown. The majority of the marker responses are below 1.5 signal to noise.

When the STEC-8 MOL-PCR assay was tested using the exclusivity panel, 80.3% of 168 possible combinations of MOLigos and template DNA returned a mean signal-to-noise ratio less than 1.5 (Figure 2). In the exclusivity panel, *E. faecalis, K. pneumoniae, M. morganii, P. rettgeri*, and *S. aureus* each contained a large number of signal-to-noise values above 1.5 for various MOL-PCR probes. In addition to the traditional Wilcoxon Exact Rank Sum test, a second condition of a signal-to-noise ratio above 1.5 was implemented into the algorithm for each data point reported from this point onward. A low signal-to-noise threshold of 1.5 was implemented to have limited impact on the statistical analysis done by the Wilcoxon test. Post two-step algorithm processing of the panel *Enterococcus faecalis, Klebsiella pneumonia, Morganella morganii Providencia rettgeri, and Staphylococcus aureus* returned STEC serogroup positive results. These 5 samples were a mix of Gram-negative and Gram-positive bacteria.

#### Evaluation of STEC reference panel

The STEC reference samples from the Moxley laboratory were analyzed with the addition of the signal-to-noise threshold along with Wilcoxon to account for any small amount of interaction with background that could be present in the purified samples. In order for a positive call to be made, both criteria (>1.5 signal-to-noise ratio and Wilcoxon critical value) were required to be met. The concentration of the reference purified STEC DNA samples tested was between 10 and 30 ng STEC DNA per reaction, which computes to 1.5 × 10^6^ to 5 × 10^6^ genomic copies per reaction. As with the initial test samples, all reactions were run in triplicate on a single plate to account for variations in reactions. The data showed (Supplemental Table 1) that the serogroup calls agreed with the known serogroups 63 out of the 79 STEC samples. The virulence gene profile data provided was incomplete for the isolates tested, i.e., presence of *stx*_1_, *stx*_2_, and *eae* was not known. Independent searches performed on the Michigan State University STEC database (www.shigatox.net) revealed incomplete data for virulence gene profiles for some strains. Literature searches were also done to obtain additional gene information missing from the original documentation provided (Stromberg et al., 2015; Toro et al., 2015). The missing data for *stx*_1_, *stx*_2_, and *eae* DNA samples in this set has been noted in Supplemental Table 1; the additional gene profile information identified has also been added. As a result of missing information, it was difficult to determine the specificity of the assay as it pertains to the virulence gene profiles for these samples. The STEC-8 MOL-PCR assay correctly identified 202 out of the 210 known virulence genes present in the 79 STEC DNA samples.

The assay identified additional serogroups along with the known serogroups for the following isolates: JB1-95, 0201 9611, MI01-88, 8266-1, and G5508, which results in a serogroup diagnostic specificity of 94% on the low end to 100% on the high end (Table 2). Included in this percentage were cases where the combined assay and algorithm returned no serogroup, when the test should have detected one of the STEC-8: MDCH-4 (excluded), KDHE 47, DEC10I (87-1713), 1:361, 1553-1, DA-37, 10C-3114 (excluded), 2002-3211, KDHE 55, 314-S, 86-24, or 403-3.

**Table 2:** **Diagnostic specificity of STEC-8 MOL-PCR assay on STEC DNA isolates**.

**Signature**	**Reference DNA Panel^a^**		**Blinded DNA Panel^b^**	
O26	97.75%	(87/89)^c^	100.00%	(115/115)
O45	97.75%	(87/89)	99.29%	(139/140)
O103	94.38%	(84/89)	96.23%	(102/106)
O104	97.75%	(87/89)	100.00%	(125/125)
O111	96.67%	(87/90)	99.12%	(113/114)
O121	94.44%	(85/90)	100.00%	(135/135)
O145	100.00%	(89/89)	100.00%	(131/131)
O157	97.75%	(87/89)	100.00%	(141/141)
*stx*_I_	86.49%	(32/37)	76.92%	(20/26)
*stx*_2_	87.50%	(49/56)	98.17%	(107/109)
*eae*	80.56%	(29/36)	96.55%	(28/29)

a Reference DNA panel from R. Moxley laboratory.

b Blinded DNA panel from T. Nagaraja laboratory.

c (m/n) ratio of true negative samples to all samples known to be negative.

MDCH-4 was originally reported as serotype O121:H19, but it was later discovered to be O113, which was corrected in Supplemental Table 1. ATCC originally reported 10C-3114 as O111:H8, and as such included that strain in its “Big-Six” non-O157 genomic DNA panel as its reference O111 serogroup. ATCC reported this was an error, and now lists 10C-3114 as serogroup O146. The MOL-PCR STEC-8 assay returned no serogroup for these two isolates, which is consistent with what was expected. The assay was able to identify a complete serogroup-virulence gene profile that matched the known profile 62 out the 79 (78.5%) STEC samples. These results demonstrated that the STEC-8 MOL-PCR assay was capable of screening samples on a large scale with the added advantage of automated data analysis.

### Evaluation of blinded samples

Further testing of the MOL-PCR assay was conducted using a set of 144 blinded samples provided by the Nagaraja laboratory (KSU). These 144 blinded samples were from supernatant lysates from pure cultures of STEC-8 strains that were boiled and centrifuge separated. Supplemental Table 2 shows a list of all 144 samples whose MOL-PCR results were sent to the laboratory at KSU for comparison to their multiplex-PCR assay (the gold standard) results; KSU identification is also provided in this list. In six of the 144 samples the assay returned more than a single serogroup. There were also instances (total of 8 of the 144) where the STEC-8 MOL-PCR assay did not return any serogroup and/or virulence gene.

Given the information that was provided about these samples (serogroup and presence of *stx*_1_, *stx*_2_, and *eae* as reported by the Nagaraja laboratory) it is possible to determine the diagnostic specificity of the STEC-8 MOL-PCR assay. There was an underrepresentation of serogroups O45 (4), O121 (9), and O157 (3) in this DNA panel. Specificity for each MOLigo pair was calculated as the percentage of the number of true negatives identified from the sum of the number of true negatives and the number of false positives, or number of actual negatives in the panel. Table 2 shows that specificity of each MOLigo pair was 76–100% depending on the MOLigo pair. The STEC-8 MOL-PCR assay identified the complete known serogroup-virulence gene profile with 86.1% (124/144) accuracy.

Table 3 shows the details of the incorrectly identified samples from both the reference set and the 144 blinded set, with the following exceptions: the exclusivity panel was not included, and if a particular sample's virulence gene was unknown then any virulence gene identified for that sample was considered correct. The STEC-8 MOL-PCR assay identified an additional serogroup 11 times and completely missed the serogroup 14 times from the combined set of 223 STEC samples. The virulence gene *eae* had the lowest number of false positives with 2 occurrences, compared to 6 for *stx*_1_ and 4 for *stx*_2_ out of the combined 223 STEC samples. The STEC-8 MOL-PCR assay missed the complete gene profile or missed one out of the three virulence genes 6 times from the 223 STEC samples.

**Table 3:** **Detailed examination of incorrect identifications**.

**Sample ID**	**Additional serogroups^a^**	**Missed serogroup^b^**	**Incorrect *stx*_1_^c^**	**Incorrect *stx*_2_^d^**	**Incorrect *eae*^e^**	**Missed gene^f^**
**STEC REFERENCE SAMPLES**						
KDHE 47		X			X	
DEC10I (87-1713)						
1:361						X
1553-1		X				
DA-37		X				X
JB1-95	X					
2002-3211		X				
KDHE 55		X			X	
314-S		X				
86-24		X				
88-1577						X
403-3		X		X		X
0201 9611	X					
MI01-88	X					
2011-5-383-1				X		
8266-1	X					
G5508	X					
**144 BLINDED STEC SAMPLES**						
Blinded 06	X					
Blinded 10	X			X		
Blinded 127		X				X
Blinded 139		X				
Blinded 14	X					
Blinded 16		X				
Blinded 21				X		
Blinded 49		X				
Blinded 70	X					
Blinded 73	X					
Blinded 74	X					
Blinded 85		X				X
Blinded 89			X			
Blinded 90			X			
Blinded 91			X			
Blinded 92			X			
Blinded 93			X			
Blinded 96		X				
Blinded 97			X			
Sum	11 of 223	14 of 223	6 of 223	4 of 223	2 of 263	6 of 223

a Additional serogroups along with the correct serogroup identified by the STEC-8 MOL-PCR assay.

b No serogroup identified by the STEC-8 MOL-PCR.

c stx_1_ identified by the STEC-8 MOL-PCR assay when it is known not to be present.

d stx_2_ identified by the STEC-8 MOL-PCR assay when it is known not to be present.

e eae identified by the STEC-8 MOL-PCR assay when it is known not to be present.

f Any virulence genes were not identified by the STEC-8 MOL-PCR assay.

## Discussion

Several nucleic acid assays are currently available for detection of STEC with multiple targets in a single reaction (Beutin et al., 2009; Bai et al., 2012; Noll et al., 2015). In many of these assays, detection of STEC is limited to a set of virulence related genes as the primary tool for early detection. The general trend displayed across many of these assays is that the virulence gene profile (e.g., *stx*_1_*, stx*_2_*, eae*) is the most important, followed by the ability to identify the STEC serogroup. There are, of course, nucleic acid assays that detect virulence genes along with STEC serogroups, but often they are run as two parallel assays (Conrad et al., 2014) and are limited in plex level (Bai et al., 2012). The benefit of detecting both serogroup and virulence gene profile in a single assay is one of monitoring samples during possible outbreaks where serogroups and virulence profiles can be used in early tracking statistics. Our STEC-8 MOL-PCR assay was capable of detecting both the serogroup of the eight important STEC serogroups as well as *stx*_1_, *stx*_2_, and *eae*, with the benefit of still being able to expand the assay to up to 50 unique DNA sequences of interest as they are identified.

Our goal for developing this STEC-8 MOL-PCR assay was to enable high-throughput, high-information content (multiplex analysis) screening of samples for STEC. The assay is intended to be utilized on samples that have already undergone enrichment culture, a standard protocol in testing labs (Gould et al., 2009) to account for problems with complex microbial backgrounds for some of the matrices along the beef production chain (e.g., fecal samples, carcass samples, hide samples, etc.). It is of greater interest that the specificity of serogroup along with virulence profile be identified. The STEC-8 MOL-PCR assay that we have described herein is capable of performing this task reliably, which adds to the ability to detect STEC in a large number of samples collected along the beef production chain. We have shown the ability to perform a single tube 11-plex assay, screening STEC-8 (O26, O45, O103, O104, O111, O121, O145, O157) along with the 3 major virulence genes (*stx*_1_, *stx*_2_, and *eae*) on as many as 24 unique samples run in triplicate in 1 day producing data with a high level of confidence.

We conducted a large-scale sample analysis with two groups of STEC and STEC associated samples. In the first reference panel set we were able to identify a complete STEC profile across the major serogroups of interest in the United States 78.5% (62/79) of the time and 86.1% (124/144) in the blinded panel. It was shown that even in cases where serogroup or gene identification was initially misrepresented, the assay was still able to make a correct identification utilizing the modified exact Wilcoxon rank sum algorithm with the assay design. It is our hope that this work will help provide useful information to the larger community of STEC research.

Every effort was made to collect a reference set of samples with a high degree of known specifications to evaluate the performance of our assay. A large number of STEC samples are readily available, but the serogroup and virulence identity for these samples is not complete or possibly misidentified. Many of the STEC strains identified have not been sequenced, which makes absolute identification difficult. These strains are generally identified using real-time multiplex PCR or multiplex PCR. Many of the commercial detection assays have a specificity that ranges from 71 to 100% (Parsons et al., 2016), which aligns with the performance of our assay at 78–86%. We believe our assay is comparable to these assays, with the added benefits of high-throughput sampling and complete serogroup-toxin profile in a single assay. Much work went into researching gene identity for isolates where this information was not known or where the reported information was in question. Literature data for some STEC isolates did not always match database values making correct identifications difficult. The MOL-PCR assay was able to identify the presence of virulence genes (*eae*) that had not been previously documented. Ten samples were identified by the assay as containing *eae*, which was later confirmed by multiplex PCR. Additionally, the assay identified blinded sample 44 (05EN000712) as having both O103 and O145, which was also confirmed.

We are currently evaluating the limit of detection for the full 11-plex STEC-8 MOL-PCR assay as well as examining the effect of complex matrices such as bovine feces in the absence of enrichment on the assay performance (Paddock et al., 2012).

Current guidance from the Centers for Disease Control and Prevention (CDC) for STEC detection and diagnosis states that human fecal samples suspect for STEC are first plated on O157 STEC selective and differential medium. Any phenotypically suspect colonies are then tested by latex agglutination for O157 antigen. If no phenotypically suspect colonies are detected, then the fecal sample is plated on less-selective medium and colonies from that plate are tested for Stx using appropriate methods, including but not limited to *stx* gene PCR. If the *stx* gene PCR, or comparable test, is confirmed positive for Stx then the sample is further characterized using PCR for *stx*_1_ and *stx*_2_ genes and at this point it is also tested for serogroups O26, O45, O103, O111, O121, O145, or O157 (Gould et al., 2009). This series of tests take a total of 2–3 days if all steps are carried out.

Clinical laboratories currently test for O157 STEC through culture, and send suspect non-O157 samples to public health laboratories, which utilize the testing process outlined above. These testing procedures are very amenable to integration of the STEC-8 MOL-PCR assay as it both identifies *stx*_1_ and *stx*_2_ as well as *eae* and each of the suggested serogroups in a single test on enriched cultures. Adding in the MOL-PCR process would be of particular benefit in cases where large-scale screening is required due to a STEC outbreak and/or food recall. It also has the benefit of reducing the total characterization time by up to 2 days by eliminating the several culturing steps currently required. Implementation of the STEC-8 MOL-PCR assay in a clinical or public health laboratory that is currently performing other PCR based assays would only require one additional piece of equipment which is essentially a standard flow cytometer.

One of the benefits to the MOL-PCR assay is the relative ease for selective modification as necessary. This assay has had a few MOLigo pair redesigns to switch certain MagPlex-TAG™ beads as well as target DNA sequence redesigns. The changes to these MOLigo pairs did not require the reaction conditions or the non-modified MOLigo pairs to be altered. If additional signatures (serogroup, toxin, etc.) of interest are identified it will be important to rapidly expand to higher level multiplexing, which is possible with the MOL-PCR assay as it will require minimal reconfiguration. The Luminex® MAGPIX® instrument is capable of testing for 50 unique signatures or markers, of which we have used only 11. We have discussed here additional components that are relevant to add to the assay, including *ehxA* (enterohemolysin) signatures and performing *eae* (Madic et al., 2010) and *stx*_2_ (Wang et al., 2002) subtyping for further characterization of samples.

Nucleic acid detection schemes like multiplex real-time PCR and other multiplex PCR technologies (the current gold standards for STEC molecular diagnostics) cannot process large sets of samples containing large amounts of information in a short period of time. This is primarily due to limitations in the information readout technology used by the process (Deshpande and White, 2012). The STEC-8 MOL-PCR assay was designed to have a robust design, which when run as described produced results with limited amounts of false positive and false negatives on 24 unique samples in single day. Reduction from triplicate sample processing to a single replicate would make it possible to process 80–88 samples for all 11 unique STEC associated signatures in single day.

## Author contributions

TW, HM, SO performed assay development. XS prepared blinded samples and performed sample validation. DM assisted with developing statistical data analysis. JB consulted on DNA signature design and optimization. RM and TN consulted on samples to process and provided STEC expertise. SG and AD guidance on assay development. All authors reviewed and edited the manuscript.

## Funding

This material is based on work that is supported by the U.S. Department of Agriculture (USDA), National Institute of Food and Agriculture under award 2012-68003-30155.

### Conflict of interest statement

The authors declare that the research was conducted in the absence of any commercial or financial relationships that could be construed as a potential conflict of interest.
