# Supplementary material for: Development of 11-Plex MOL-PCR Assay for the Rapid Screening of Samples for Shiga Toxin-Producing Escherichia coli
Source: Front Cell Infect Microbiol. 2016 Aug 31;6:92. doi: 10.3389/fcimb.2016.00092 (PMC5005322; doi:10.3389/fcimb.2016.00092)
Supplement: Supplementary file 1 [file Table1.DOCX]

| Supplemental Table 1  Comparison of STEC-8 MOL-PCR assay to known database information for serogroup and virulence profile for reference isolates | | | | |
| --- | --- | --- | --- | --- |
|  | MOL-PCR Assay^1^ / Database Values^2^ | | | |
| Isolate | **Serogroup** | ***stx_1_*** | ***stx_2_*** | ***eae*** |
| CDC 97-3068 | O121 / O121 | -/- | +/+ | +/+ |
| MDCH-4 | - / O113 | -/*^3^ | +/+ | -/- |
| 2000-3039 | O45 / O45 | +/+ | -/- | +/+ |
| 8-084 | O121 / O121 | -/* | +/* | +/* |
| 10049 | O111 / O111 | +/+ | -/- | +/+ |
| 83-75 | O145 / O145 | -/- | +/+ | +/+ |
| B8026-C1 | O45 / O45 | +/+ | -/- | +/+ |
| 236-1 | O103 / O103 | +/+ | +/+ | +/+ |
| MT#2 | O121 / O121 | -/- | +/+ | +/+ |
| 2011-0-1256 | O104 / O104 | +/+ | -/- | -/- |
| KDHE 47 | - / O121 | -/* | +/+ | +/- |
| TB154A | O103 / O103 | +/+ | -/- | +/+ |
| S2006 #1 | O157 / O157 | +/+ | +/+ | +/+ |
| DA-21 | O45 / O45 | +/+ | -/- | +/+ |
| 2006-3008 | O103 / O103 | +/+ | -/* | +/+ |
| DEC11C | O45 / O45 | +/+ | -/- | +/+ |
| IHIT2087 | O26 / O26 | +/+ | -/* | +/+ |
| 3215-99 (F6627) | O111 / O111 | +/+ | +/+ | +/+ |
| B2387 | O157 / O157 | -/* | +/+ | +/* |
| DEC10I (87-1713) | - / O145 | +/+ | -/- | +/+ |
| 1234-1 | O145 / O145 | +/+ | +/+ | +/+ |
| DEC10E | O26/ O26 | +/+ | -/- | +/+ |
| B8227-C8 | O45 / O45 | +/+ | -/- | +/+ |
| 7726-1 | O111 / O111 | +/+ | +/+ | +/+ |
| TY-2482 | O104 / O104 | -/- | +/+ | -/- |
| B6820-C1 | O145 / O145 | +/* | +/+ | +/+ |
| MI-0041B | O104 / O104 | -/* | +/* | -/* |
| 1.2622 | O45 / O45 | +/+ | -/- | -/- |
| IH 16 | O145 / O145 | -/- | +/+ | +/+ |
| S2006 #4 | O157 / O157 | +/+ | +/+ | +/+ |
| 15612-1 | O103 / O103 | +/+ | -/- | +/+ |
| 236-5 | O103 / O103 | +/+ | +/+ | +/+ |
| 99-3311 | O145 / O145 | +/+ | +/+ | +/+ |
| 1:361 | - / O157 | -/- | +/+ | -/+ |
| B8228-C2 | O45 / O45 | +/+ | -/- | +/* |
| RD8 (7075) | O111 / O111 | -/- | +/+ | -/- |
| 1553-1 | - / O121 | +/+ | +/- | -/- |
| CDC 1994 3023 | O104 / O104 | -/- | +/+ | -/- |
| H30 | O26 / O26 | +/+ | -/- | +/+ |
| DA-37 | - / O121 | -/- | +/+ | -/+ |
| 2003-3014 | O26 / O26 | +/+ | +/+ | +/+ |
| JB1-95 | O45, O111 / O111 | +/+ | +/+ | +/+ |
| IHIT1703 | O111 / O111 | +/+ | -/- | +/+ |
| DEC8b | O111 / O111 | +/+ | +/+ | +/+ |
| S2006 #2 | O157 / O157 | +/+ | +/+ | +/+ |
| 10C-3114 | - / O146 | -/* | +/+ | -/- |
| IHIT0304 | O145 / O145 | -/- | +/+ | +/+ |
| 93-111 | O157 / O157 | +/+ | +/+ | +/+ |
| MT (CDC 1994 3024) | O104 / O104 | -/- | +/+ | -/- |
| 2002-3211 | - / O121 | -/- | +/+ | +/+ |
| CDC 96-3285 | O45 / O45 | +/+ | -/- | +/+ |
| 8419 | O103 / O103 | +/+ | -/- | +/+ |
| DA-10 | O26 / O26 | +/+ | -/- | +/+ |
| 97-3250 | O26 / O26 | +/+ | +/+ | +/+ |
| GS G5578620 | O145 / O145 | +/+ | -/- | +/+ |
| KDHE 55 | - / O121 | -/- | +/+ | +/+ |
| 7744 | O145 / O145 | +/+ | -/- | +/+ |
| CDC 90-3128 | O103 / O103 | +/+ | -/- | +/+ |
| 89-118 | O103 / O103 | +/* | -/* | +/* |
| 933 | O157 / O157 | +/+ | +/+ | +/+ |
| 314-S | - / O145 | +/+ | -/- | +/+ |
| MT#80 | O103 / O103 | +/+ | -/- | +/+ |
| M535 | O104 / O104 | +/+ | +/+ | -/* |
| S2006 #3 | O157 / O157 | +/+ | +/+ | +/+ |
| 413/89-1 | O26 / O26 | +/+ | -/- | +/+ |
| 86-24 | - / O157 | -/- | +/+ | +/+ |
| DEC10B | O26 / O26 | +/+ | -/- | +/+ |
| 16272 | O26 / O26 | +/+ | -/- | +/+ |
| 88-1577 | O26 / O26 | -/+ | -/- | +/+ |
| D88-28058 | O45 / O45 | +/* | -/* | +/* |
| 403-3 | - / O121 | -/+ | +/- | -/* |
| 9:100 | O157 / O157 | -/- | +/+ | +/+ |
| RW1372 | O103 / O103 | +/+ | -/- | +/+ |
| 0201 9611 | O26, O103, O111 / O111 | +/+ | -/- | +/+ |
| MI01-88 | O45, O121 / O45 | +/+ | -/- | +/+ |
| 2011-5-383-1 | O104 / O104 | +/+ | +/- | -/- |
| 8266-1 | O103, O111 / O111 | +/+ | +/+ | +/+ |
| G5508 | O26, O104 / O104 | -/- | +/+ | -/* |
| MDCH_male_ 069311 | O104 / O104 | -/* | +/* | -/* |
| G58-1 | - / O101 | -/- | -/- | -/- |
| 2534-86 | - / O8 | -/- | -/- | -/- |
| 11182-2 | - / O142 | +/+ | -/- | -/- |
| 16118-2 | - / O142 | +/+ | -/- | -/- |
| ECOR 26 | O104 / O104 | -/- | -/- | -/- |
| 43893 | - / O124 | -/- | -/- | -/- |

| ^1^Fields are marked with the serogroup name or “+” that were statistically determined to be present; fields marked with “-” denote no serogroup or genes were determined to be present  ^2^Fields are marked with the known database values for the samples as provided from collaborators; fields marked with “-” are known to be negative for that gene  ^3^ Fields are marked with “*” to denote data for virulence gene was not provided or found in literature searches |
| --- |
