# Supplementary material for: Development of 11-Plex MOL-PCR Assay for the Rapid Screening of Samples for Shiga Toxin-Producing Escherichia coli
Source: Front Cell Infect Microbiol. 2016 Aug 31;6:92. doi: 10.3389/fcimb.2016.00092 (PMC5005322; doi:10.3389/fcimb.2016.00092)
Supplement: Supplementary file 2 [file Table2.docx]

|  | | | | | | |  |  |
| --- | --- | --- | --- | --- | --- | --- | --- | --- |
| **Supplemental Table 2** Comparison of STEC-8 MOL-PCR assay to known database information for serogroup and virulence profile for blinded isolates | | | | | | |  |  |
|  | MOL-PCR Assay^1^ / Database Values^2^ | | | |  |  |  |  |
| **Sample ID** | **Serotype** | ***stx*_1_** | ***stx*_2_** | ***eae*** | **Strain ID** |  |  |  |
| Blinded 01 | O26 / O26 | -/- | -/- | +/+ | DEC 9f |  |  |  |
| Blinded 02 | O111 / O111 | +/+ | -/- | +/+ | 86-10049 |  |  |  |
| Blinded 03 | O26 / O26 | +/+ | -/- | +/+ | DEC 10e |  |  |  |
| Blinded 04 | O145 / O145 | -/- | +/+ | +/+ | B6820-C1 |  |  |  |
| Blinded 05 | O26 / O26 | +/+ | -/- | +/+ | TB285A |  |  |  |
| Blinded 06 | O26, O103 / O26 | +/+ | -/- | +/+ | TB285C |  |  |  |
| Blinded 07 | O145 / O145 | -/- | -/- | +/+ | BCL73 |  |  |  |
| Blinded 08 | O103 / O103 | +/+ | +/+ | +/+ | 236-5 |  |  |  |
| Blinded 09 | O103 / O103 | +/+ | +/+ | +/+ | 236-1 |  |  |  |
| Blinded 10 | O103, O111 / O111 | +/+ | +/- | +/+ | ED-47 |  |  |  |
| Blinded 11 | O111 / O111 | -/- | +/+ | -/- | RD8 |  |  |  |
| Blinded 12 | O111 / O111 | +/+ | +/+ | +/+ | 3007-85 |  |  |  |
| Blinded 13 | O26 / O26 | -/- | -/- | +/+ | LTO26-12 |  |  |  |
| Blinded 14 | O26, O103 / O26 | -/- | -/- | +/+ | LTO26-17 |  |  |  |
| Blinded 15 | O26 / O26 | -/- | -/- | +/+ | LTO26-19 |  |  |  |
| Blinded 16 | - / O111 | +/+ | +/+ | +/+ | W29104 |  |  |  |
| Blinded 17 | O103 / O103 | +/+ | -/- | +/+ | 6:38 |  |  |  |
| Blinded 18 | O26 / O26 | +/+ | +/+ | +/+ | 10.253 |  |  |  |
| Blinded 19 | O26 / O26 | +/+ | -/- | +/+ | 413/89-1 |  |  |  |
| Blinded 20 | O145 / O145 | -/- | +/+ | +/+ | IHIT0304 |  |  |  |
| Blinded 21 | O26 / O26 | +/+ | +/- | +/+ | IHIT2087 |  |  |  |
| Blinded 22 | O103 / O103 | +/+ | -/- | +/+ | RW1372 |  |  |  |
| Blinded 23 | O103 / O103 | +/+ | -/- | +/+ | RW1374 |  |  |  |
| Blinded 24 | O103 / O103 | +/+ | -/- | +/+ | PMK-5 |  |  |  |
| Blinded 25 | O26 / O26 | +/+ | -/- | +/+ | DA-22 |  |  |  |
| Blinded 26 | O103 / O103 | +/+ | -/- | +/+ | DA-34 |  |  |  |
| Blinded 27 | O103 / O103 | +/+ | -/- | +/+ | TB154A |  |  |  |
| Blinded 28 | O145 / O145 | +/+ | -/- | +/+ | MT#66 |  |  |  |
| Blinded 29 | O103 / O103 | +/+ | -/- | +/+ | MT#82 |  |  |  |
| Blinded 30 | O26 / O26 | -/- | +/+ | +/+ | CB7776 |  |  |  |
| Blinded 31 | O26 / O26 | -/- | +/+ | +/+ | CB8240 |  |  |  |
| Blinded 32 | O26 / O26 | +/+ | +/+ | +/+ | CB8474 |  |  |  |
| Blinded 33 | O26 / O26 | +/+ | -/- | +/+ | EK29 |  |  |  |
| Blinded 34 | O103 / O103 | +/+ | -/- | +/+ | EK30 |  |  |  |
| Blinded 35 | O103 / O103 | +/+ | -/- | +/+ | EK32 |  |  |  |
| Blinded 36 | O111 / O111 | +/+ | -/- | +/+ | EK34 |  |  |  |
| Blinded 37 | O111 / O111 | +/+ | -/- | +/+ | EK35 |  |  |  |
| Blinded 38 | O103 / O103 | +/+ | -/- | +/+ | 00-3308 |  |  |  |
| Blinded 39 | O103 / O103 | +/+ | -/- | +/+ | 01-3224 |  |  |  |
| Blinded 40 | O145 / O145 | -/- | -/- | +/+ | 02-3422 |  |  |  |
| Blinded 41 | O145 / O145 | -/- | +/+ | +/+ | IH 16 |  |  |  |
| Blinded 42 | O26 / O26 | +/+ | -/- | +/+ | IH 19 |  |  |  |
| Blinded 43 | O145 / O145 | -/- | +/+ | +/+ | 4865/96 |  |  |  |
| Blinded 44 | O103, O145 / O103, O145 | +/+ | +/+ | +/+ | 05EN000712 |  |  |  |
| Blinded 45 | O26 / O26 | +/+ | -/- | +/+ | H19 |  |  |  |
| Blinded 46 | O157 / O157 | -/- | +/+ | +/+ | CDC B1409-C1 |  |  |  |
| Blinded 47 | O157 / O157 | +/+ | -/- | +/+ | CDC C984 |  |  |  |
| Blinded 48 | O157 / O157 | +/+ | +/+ | +/+ | CDC EDL 932 |  |  |  |
| Blinded 49 | - / O104 | -/- | +/+ | -/- | MT |  |  |  |
| Blinded 50 | O104 / O104 | +/+ | -/- | -/- | 2011-5-383-1 |  |  |  |
| Blinded 51 | O104 / O104 | +/+ | -/- | -/- | 2011-0-1256 |  |  |  |
| Blinded 52 | O45 / O45 | -/- | -/- | -/- | 88.1622 |  |  |  |
| Blinded 53 | O45 / O45 | +/+ | -/- | -/- | 1.2622 |  |  |  |
| Blinded 54 | O45 / O45 | -/- | -/- | +/+ | 2566-58 |  |  |  |
| Blinded 55 | O104 / O104 | +/+ | -/- | -/- | 2013-6-48C |  |  |  |
| Blinded 56 | O104 / O104 | +/+ | -/- | -/- | 2013-6-122E |  |  |  |
| Blinded 57 | O104 / O104 | +/+ | -/- | -/- | 2013-6-148B |  |  |  |
| Blinded 58 | O104 / O104 | +/+ | -/- | -/- | 2013-6-658A |  |  |  |
| Blinded 59 | O104 / O104 | +/+ | -/- | -/- | 2013-6-666C |  |  |  |
| Blinded 60 | O104 / O104 | +/+ | -/- | -/- | 2013-6-660A |  |  |  |
| Blinded 61 | O104 / O104 | +/+ | -/- | -/- | 2013-6-659D |  |  |  |
| Blinded 62 | O104 / O104 | +/+ | -/- | -/- | 2013-6-664C |  |  |  |
| Blinded 63 | O104 / O104 | +/+ | -/- | -/- | 2013-6-669B |  |  |  |
| Blinded 64 | O104 / O104 | +/+ | -/- | -/- | 2013-6-672E |  |  |  |
| Blinded 65 | O104 / O104 | +/+ | -/- | -/- | 2013-6-675F |  |  |  |
| Blinded 66 | O104 / O104 | +/+ | -/- | -/- | 2013-6-678F |  |  |  |
| Blinded 67 | O104 / O104 | +/+ | -/- | -/- | 2013-6-684C |  |  |  |
| Blinded 68 | O104 / O104 | +/+ | -/- | -/- | 2013-6-685A |  |  |  |
| Blinded 69 | O104 / O104 | +/+ | -/- | -/- | 2013-6-691A |  |  |  |
| Blinded 70 | O103, O104 / O104 | +/+ | -/- | -/- | 2013-6-665E |  |  |  |
| Blinded 71 | O103 / O103 | +/+ | -/- | +/+ | 3720-1 |  |  |  |
| Blinded 72 | O103 / O103 | +/+ | -/- | +/+ | 9012-1 |  |  |  |
| Blinded 73 | O45, O103 / O103 | +/+ | -/- | +/+ | 10674-1 |  |  |  |
| Blinded 74 | O103, O111 / O103 | +/+ | -/- | +/+ | 15612-1 |  |  |  |
| Blinded 75 | O103 / O103 | +/+ | -/- | +/+ | 15687-1 |  |  |  |
| Blinded 76 | O103 / O103 | +/+ | -/- | +/+ | 18380-2 |  |  |  |
| Blinded 77 | O111 / O111 | +/+ | -/- | +/+ | 1939 |  |  |  |
| Blinded 78 | O111 / O111 | +/+ | +/+ | +/+ | 7726-1 |  |  |  |
| Blinded 79 | O111 / O111 | +/+ | +/+ | +/+ | 7728-1 |  |  |  |
| Blinded 80 | O111 / O111 | +/+ | +/+ | +/+ | 7730 |  |  |  |
| Blinded 81 | O111 / O111 | +/+ | +/+ | +/+ | 7732-1 |  |  |  |
| Blinded 82 | O111 / O111 | +/+ | +/+ | +/+ | 7734-1 |  |  |  |
| Blinded 83 | O111 / O111 | +/+ | +/+ | +/+ | 7739-1 |  |  |  |
| Blinded 84 | O111 / O111 | +/+ | +/+ | +/+ | 7740-1 |  |  |  |
| Blinded 85 | - / O111 | -/+ | -/+ | -/+ | 7756-1 |  |  |  |
| Blinded 86 | O111 / O111 | +/+ | +/+ | +/+ | 7766-1 |  |  |  |
| Blinded 87 | O111 / O111 | +/+ | +/+ | +/+ | 8266-1 |  |  |  |
| Blinded 88 | O111 / O111 | +/+ | -/- | +/+ | 11189-1 |  |  |  |
| Blinded 89 | O121 / O121 | +/- | -/- | -/- | 1553-1 |  |  |  |
| Blinded 90 | O121 / O121 | +/- | -/- | -/- | 1864 |  |  |  |
| Blinded 91 | O121 / O121 | +/- | -/- | -/- | 4188-1 |  |  |  |
| Blinded 92 | O121 / O121 | +/- | -/- | -/- | 7236 |  |  |  |
| Blinded 93 | O121 / O121 | +/- | -/- | -/- | 18698-1 |  |  |  |
| Blinded 94 | O145 / O145 | +/+ | +/+ | +/+ | 1234-1 |  |  |  |
| Blinded 95 | O145 / O145 | +/+ | -/- | +/+ | 7744 |  |  |  |
| Blinded 96 | - / O145 | +/+ | -/- | +/+ | 13478-1 |  |  |  |
| Blinded 97 | O121 / O121 | +/- | -/- | -/- | 4190 |  |  |  |
| Blinded 98 | O145 / O145 | +/+ | -/- | +/+ | KDHE 1 |  |  |  |
| Blinded 99 | O103 / O103 | +/+ | -/- | +/+ | KDHE 2 |  |  |  |
| Blinded 100 | O26 / O26 | +/+ | -/- | +/+ | KDHE 3 |  |  |  |
| Blinded 101 | O111 / O111 | +/+ | +/+ | +/+ | KDHE 4 |  |  |  |
| Blinded 102 | O26 / O26 | +/+ | -/- | +/+ | KDHE 6 |  |  |  |
| Blinded 103 | O26 / O26 | +/+ | -/- | +/+ | KDHE 7 |  |  |  |
| Blinded 104 | O111 / O111 | +/+ | +/+ | +/+ | KDHE 8 |  |  |  |
| Blinded 105 | O26 / O26 | +/+ | -/- | +/+ | KDHE 9 |  |  |  |
| Blinded 106 | O26 / O26 | +/+ | -/- | +/+ | KDHE 10 |  |  |  |
| Blinded 107 | O103 / O103 | +/+ | -/- | +/+ | KDHE 11 |  |  |  |
| Blinded 108 | O26 / O26 | +/+ | -/- | +/+ | KDHE 12 |  |  |  |
| Blinded 109 | O26 / O26 | +/+ | -/- | +/+ | KDHE 13 |  |  |  |
| Blinded 110 | O111 / O111 | +/+ | -/- | +/+ | KDHE 15 |  |  |  |
| Blinded 111 | O103 / O103 | +/+ | -/- | +/+ | KDHE 16 |  |  |  |
| Blinded 112 | O103 / O103 | +/+ | -/- | +/+ | KDHE 18 |  |  |  |
| Blinded 113 | O111 / O111 | +/+ | -/- | +/+ | KDHE 19 |  |  |  |
| Blinded 114 | O103 / O103 | +/+ | -/- | +/+ | KDHE 20 |  |  |  |
| Blinded 115 | O111 / O111 | +/+ | -/- | +/+ | KDHE 21 |  |  |  |
| Blinded 116 | O45 / O45 | +/+ | -/- | +/+ | KDHE 22 |  |  |  |
| Blinded 117 | O103 / O103 | +/+ | -/- | +/+ | KDHE 23 |  |  |  |
| Blinded 118 | O103 / O103 | +/+ | -/- | +/+ | KDHE 25 |  |  |  |
| Blinded 119 | O103 / O103 | +/+ | -/- | +/+ | KDHE 28 |  |  |  |
| Blinded 120 | O26 / O26 | +/+ | -/- | +/+ | KDHE 29 |  |  |  |
| Blinded 121 | O26 / O26 | +/+ | -/- | +/+ | KDHE 30 |  |  |  |
| Blinded 122 | O111 / O111 | +/+ | +/+ | +/+ | KDHE 31 |  |  |  |
| Blinded 123 | O103 / O103 | +/+ | -/- | +/+ | KDHE 32 |  |  |  |
| Blinded 124 | O103 / O103 | +/+ | -/- | +/+ | KDHE 35 |  |  |  |
| Blinded 125 | O103 / O103 | +/+ | -/- | +/+ | KDHE 38 |  |  |  |
| Blinded 126 | O103 / O103 | +/+ | -/- | +/+ | KDHE 39 |  |  |  |
| Blinded 127 | - / O103 | -/+ | -/- | -/+ | KDHE 40 |  |  |  |
| Blinded 128 | O103 / O103 | +/+ | -/- | +/+ | KDHE 41 |  |  |  |
| Blinded 129 | O111 / O111 | +/+ | -/- | +/+ | KDHE 43 |  |  |  |
| Blinded 130 | O103 / O103 | +/+ | -/- | +/+ | KDHE 44 |  |  |  |
| Blinded 131 | O26 / O26 | +/+ | -/- | +/+ | KDHE 46 |  |  |  |
| Blinded 132 | O121 / O121 | -/- | +/+ | +/+ | KDHE 47 |  |  |  |
| Blinded 133 | O121 / O121 | -/- | +/+ | +/+ | KDHE 48 |  |  |  |
| Blinded 134 | O111 / O111 | +/+ | -/- | +/+ | KDHE 49 |  |  |  |
| Blinded 135 | O26 / O26 | +/+ | -/- | +/+ | KDHE 50 |  |  |  |
| Blinded 136 | O111 / O111 | +/+ | -/- | +/+ | KDHE 51 |  |  |  |
| Blinded 137 | O145 / O145 | +/+ | +/+ | +/+ | KDHE 53 |  |  |  |
| Blinded 138 | O103 / O103 | +/+ | -/- | +/+ | KDHE 54 |  |  |  |
| Blinded 139 | - / O121 | -/- | +/+ | +/- | KDHE 55 |  |  |  |
| Blinded 140 | O103 / O103 | +/+ | -/- | +/+ | KDHE 56 |  |  |  |
| Blinded 141 | O111 / O111 | +/+ | -/- | +/+ | KDHE 57 |  |  |  |
| Blinded 142 | O111 / O111 | +/+ | -/- | +/+ | KDHE 58 |  |  |  |
| Blinded 143 | O103 / O103 | +/+ | -/- | +/+ | KDHE 59 |  |  |  |
| Blinded 144 | O26 / O26 | +/+ | -/- | +/+ | KDHE 60 |  |  |  |
| ^1^Fields are marked with the serogroup name or “+” that were statistically determined to be present; fields marked with “-” denote no serogroup or genes were determined to be present | | | | | | | |  |
| ^2^Fields are marked with the known database values for the samples as provided from collaborators; fields marked with “-” are known to be negative for that gene | | | | | | | |  |
